# Curriculum and Competency Guidelines for the Advanced Care Practitioner in Infectious Disease

**DOI:** 10.1093/ofid/ofad589

**Published:** 2024-01-31

**Authors:** Miriam A Smith, Paul Zelenetz, Angela Kim, Henry Donaghy, J Scott Gould, Renee McLeod-Sordjan

**Affiliations:** Daytonand Karen Brown Division of Infectious Diseases, Donald and Barbara Zucker School of Medicine at Hofstra/Northwell, Manhasset, New York, USA; Department of Medicine, Donald and Barbara Zucker School of Medicine at Hofstra/Northwell, Manhasset, NewYork, USA; Daytonand Karen Brown Division of Infectious Diseases, Donald and Barbara Zucker School of Medicine at Hofstra/Northwell, Manhasset, New York, USA; Department of Medicine, Donald and Barbara Zucker School of Medicine at Hofstra/Northwell, Manhasset, NewYork, USA; Daytonand Karen Brown Division of Infectious Diseases, Donald and Barbara Zucker School of Medicine at Hofstra/Northwell, Manhasset, New York, USA; Department of Medicine, Donald and Barbara Zucker School of Medicine at Hofstra/Northwell, Manhasset, NewYork, USA; Daytonand Karen Brown Division of Infectious Diseases, Donald and Barbara Zucker School of Medicine at Hofstra/Northwell, Manhasset, New York, USA; Department of Medicine, Donald and Barbara Zucker School of Medicine at Hofstra/Northwell, Manhasset, NewYork, USA; Hofstra/Northwell School of Nursing and Physician Assistant Studies, 160 Hofstra University, Hempstead, NewYork, USA; Hofstra/Northwell School of Nursing and Physician Assistant Studies, 160 Hofstra University, Hempstead, NewYork, USA

**Keywords:** ACP, ID subspecialty ACP, nurse practitioner, physician assistant

## Abstract

**Background:**

Changes in the health care delivery system have altered the way internal medicine (IM) is practiced, with inclusion of subspecialty advanced care practitioners (ACPs) as vital members of the health care team.

**Methods:**

ACPs are provided the clinical settings and educational resources within an academic center to become competent in recognizing and managing common and complicated infectious diseases (ID). The ID ACP will be given progressive responsibility with expectations for achievement of milestones as they develop into competent practitioners. We seek to ensure quality, cost-effective, and comprehensive patient-centered care on the ID service in the inpatient and ambulatory settings in compliance with national standards and scope of practice recommendations and regulations.

**Results:**

In recognition of the expanding role of ACPs, we developed a curriculum and guidelines in the subspecialty of ID.

**Conclusions:**

Our proposal greatly adds to the available literature for ACPs to provide the full spectrum of ID practice.

## Teaser

We have developed a curriculum and competency guidelines for Advanced Care Practitioners in Infectious Disease (ID). Our document provides the foundation for the full scope of ID practice with emphasis on clinical experience as the principal means of achieving competence.

Internal medicine and its subspecialties account for the largest number of practicing physicians. Despite this, changes in the health care delivery system have altered the way in which IM is practiced, resulting in an increased reliance on nonphysician practitioners (NPPs).

NPPs are accredited providers who provide health care either under the guidance of or in collaboration with physicians. The Centers for Medicare and Medicaid Services (CMS) recognize both physicians and NPPs as providers of health care across diverse settings. NPPs include physician assistants (PAs), nurse practitioners (NPs), clinical nurse specialists, certified nurse midwives, and certified registered nurse anesthetists [1]. For the purposes of this article, we are focusing on NPs and PAs as the subset of NPPs who will be referred to collectively as advanced care practitioners (ACPs).

The adoption of the 6 core competencies by the Accreditation Council for Graduate Medical Education (ACGME) and the American Board of Medical Specialties (ABMS) provides the foundation for outcomes-based undergraduate and graduate medical education, board certification, and maintenance of certification, as well as an unprecedented opportunity to create a seamless continuum of learning and assessment in medicine [2–5].

In recognition of the expanding role of ACPs, we have developed a curriculum and guideline competency for nurse practitioners (NPs) and physician assistants (PAs) in the subspecialty of infectious diseases (ID). We used an interprofessional, competency-based education (CBE) framework incorporating 6 core competencies to assess EPA development [2–7]. Our proposal is based on recommendations and/or proposed guidelines from regulatory agencies, task force educators, and professional organizations representing IM, nursing, and PAs [2–5, 8–18]. The 6 core competencies serve as the basis for developing our curriculum and competency guideline proposal for ACPs in ID.

Academic centers with an interest in promoting education for ACPs also have training programs for medical residents and fellows. ACPs have an impact on resident workload, continuity of care, and patient coordination education [19–22]. Surveys of medical trainees and program directors (PDs) report that adding ACPs positively affects physician medical training [20, 21]. Integration of well-trained ACPs with residents encourages resident leadership development, enhances service/education balance, and promotes interprofessional collaboration, thereby strengthening the academic practice partnership [22–24]. The impact of ACP inclusion in subspecialty practice on resident and fellow training at academic medical centers will be important to optimize with respect to the education of physicians in training while also promoting career development of ACPs seeking better preparation for practice in specialized areas of medicine. We foresee a healthy, ongoing collaboration between physician and nonphysician practitioners in the health care environment extending well beyond the training period.

Our ACPs are provided clinical settings and educational resources as part of Northwell Health, a large health care system in the NYC metropolitan area comprising 23 hospitals and many more ambulatory practices. The Donald and Barbara Zucker School of Medicine at Hofstra/Northwell opened in 2011 to its first medical school class. Since then, Hofstra University has expanded its PA program and opened the Hofstra-Northwell School of Nursing, graduating its students with Master’s and doctoral degrees.

As the practice of ID encompasses a wide variety of illnesses, ACPs in ID should become competent in the recognition and management of common and complicated infectious diseases, the latter with concern for clinical deterioration and potential life-threatening illness. The ID ACP should understand the principles of identifying microorganisms and antimicrobial susceptibility testing, interpret results of microbiologic, serologic, and molecular testing, determine appropriate use and implementation of antimicrobial agents and biologics, and develop a foundation of knowledge regarding the principles of health epidemiology, infection control, and immunization. The ACP will be given progressive degrees of responsibility, with expectations for achievement of milestone competencies.

Numerous PA and joint PA/NP postgraduate training programs are available in a variety of specialties [25]. However, significant heterogeneity exists among competency frameworks as described by Kesten and Beebe for NP education and training programs [26]. There is a growing body of literature addressing education initiatives and competency standards for trainees [2, 4, 8, 11–18] but a paucity of literature addressing curriculum and competency for ACPs who are now in subspecialty practice. To our knowledge, there are no curriculum or competency guidelines for ACPs to provide the full spectrum of ID practice.

As the role for ACPs continues to expand, we convened a working group of health system leaders to develop curriculum and competency guidelines for NPs and PAs who seek to practice as subspecialists in ID in compliance with national standards and regulations that govern ACP-licensed practitioners. This working group included representation from NP, PA, and physician medical education as well as content experts from Northwell Health's Division of ID. The current proposal is based on an interprofessional approach utilizing the ACGME residency and fellowship training model, AACN competency standards, revised competency standards for PAs, and recommendations and/or proposed guidelines from regulatory agencies, task force educators, and professional organizations representing IM/ID, nursing, and PAs [2–5, 8–18]. Our document also provides a template for using competencies in assessing EPAs in practice. Our proposed curriculum and guidelines detail and emphasize the clinical time required to achieve mastery and greatly expand the available literature for ACP subspecialists who have completed their training and have entered an ID subspecialty practice setting.

## GOALS AND OBJECTIVES FOR SUBSPECIALTY ACPS

Our proposed curriculum and competency guideline document includes goals and objectives for subspecialty ACPs based on 6 core competencies: patient care, medical knowledge, practice-based learning and environment, interpersonal and communication skills, professionalism, and systems-based practice [2–5, 8–18]. Subspecialty ACPs in ID are expected to (1) provide patient care that is compassionate, appropriate, and effective for the promotion of health, prevention of illness, and treatment of infectious diseases; (2) demonstrate knowledge of established and evolving biomedical, clinical, and social sciences and be able to apply their knowledge to patient care and the education of others in the area of infectious disease; (3) use scientific evidence and methods to investigate, evaluate, and improve patient care practices in the subspecialty; (4) demonstrate interpersonal and communication skills that enable them to establish and maintain professional relationships with patients, families, and other members of health care teams; (5) demonstrate behaviors that reflect a commitment to continuous professional development and ethical practice, as well as an understanding and sensitivity to diversity and a responsible attitude toward their patients, their profession, and society; and (6) demonstrate both an understanding of the contexts and systems in which health care is provided and the ability to apply this knowledge to optimize health care [9].

We present a timeline describing the ACP clinical experience, progression of responsibility, and expectation of milestone achievement by the end of 1 year in accordance with regulatory requirements for scope of practice under attending physician supervision (Table 1) [2–4].

**Table 1.** Core Competencies (Inpatient and/or Ambulatory Settings for the ACP Subspecialist in ID)

I. Patient Care		
By the end of the first 3 months and under the supervision of the ID attending, the ACP will be expected to:	By the end of the first 6 months and under the supervision of the ID attending, the ACP will be expected to:	By the end of the 1st year and under the supervision of the ID attending, the ACP will be expected to:
Provide new ID consultations for at least 2 patients daily and follow up on 3 additional patients daily Acquire accurate and relevant history from the patient and/or medical record in a prioritized and hypothesis-driven fashion Seek and obtain appropriate, verified, and prioritized data from secondary sources (ie, family, prior medical records, pharmacy) Perform an accurate physical examination targeted to the patient's complaints and medical conditions Differentiate abnormal from normal examination findings Understand the physical examination findings in the context of the patient's complaints/medical conditions Track important changes in the physical examination over time Synthesize all available data, including interview, physical examination, and preliminary laboratory data, to define each patient's central clinical problem Begin to manage patients with common clinical disorders in ID described in the “knowledge section” Make appropriate decisions/recommendations based on the results of common diagnostic testing, including but not limited to routine blood chemistries, hematologic studies, coagulation tests, arterial blood gases, EKG, chest radiographs, urinalysis and analysis of other body fluids, reflecting clinical reasoning skills Recognize when to seek additional guidance	Provide new ID consultations for at least 3 patients daily and follow up on 6 additional patients daily Identify subtle or unusual physical findings that may influence clinical decision-making Develop prioritized differential diagnoses and evidence-based diagnostic/therapeutic plans for common ID conditions Make appropriate clinical decisions based on results of more advanced diagnostic tests Customize care in the context of the patient's preferences and overall health Recognize when to seek additional guidance	Provide new ID consultations for at least 4 patients daily and almost independently follow up on 8 additional patients daily Identify subtle or unusual physical findings that may influence clinical decision-making Develop prioritized differential diagnoses and evidence-based diagnostic/therapeutic plans for *more complex* ID conditions Make appropriate clinical decisions based on the results of more advanced diagnostic tests Customize care in the context of the patient's preferences and overall health Recognize when to seek additional guidance

II. Medical Knowledge	
By the end of 6 months, the ID subspecialist ACP will be expected to:	By the end of year 1, the ID subspecialist ACP will also be expected to:
Understand the relevant pathophysiology and basic science for common ID conditions Develop an understanding of basic biostatistics terminology and application (ie, sensitivity, specificity, PPV, NPV) Demonstrate sufficient knowledge to evaluate common ID conditions Understand indications for and basic interpretation of common diagnostic testing including but not limited to routine blood chemistries, hematologic studies, coagulation tests, ABGs, EKGs, CXRs, PFTs, urinalysis, and other body fluid analyses Have command of performing histories and physicals, the differential thought process, therapeutics, and follow-up of chronic medical conditions	Demonstrate sufficient knowledge to evaluate complex ID conditions and multiple coexistent conditions Understand the relevant pathophysiology and basic science for uncommon or complex ID conditions Understand indications for and develop basic skills in interpreting more advanced diagnostic tests

III. Practice-Based Learning and Improvement	
By the end of 6 months, the ID subspecialist ACP will be expected to:	By the end of year 1, the ID subspecialist ACP will also be expected to:
Identify strengths, deficiencies, and limits in one's knowledge and expertise Set learning and improvement goals Identify learning needs as they emerge in patient care activities Precisely articulate clinical questions Access medical information resources to answer clinical questions and support decision-making Effectively and efficiently search databases for original clinical research articles Effectively and efficiently search evidence-based summary medical information resources Determine if clinical evidence can be generalized to an individual patient Respond welcomingly and productively to feedback from all members of the health care team	Systematically analyze practice using quality improvement methods and implement changes with the goal of practice improvement Incorporate formative evaluation feedback into daily practice Locate, appraise, and assimilate evidence from scientific studies related to their patients’ ID-related problems Use information technology to optimize learning Reflect on audit compared with local or national benchmarks; explore possible explanations for deficiencies including doctor-related, system-related, and patient-related factors Identify areas in practice and local system that can be changed to improve processes and outcomes of care Engage in quality improvement intervention Appraise the quality of medical information/clinical guideline resources and application to the clinical question Integrate clinical evidence, clinical context, and patient preferences into decision-making

IV. Systems-Based Practice	
By the end of 6 months, the ID subspecialist ACP will be expected to:	By the end of year 1, the ID subspecialist ACP will also be expected to:
Appreciate roles of a variety of health care providers, including but not limited to consultants, therapists, nurses, home care workers, pharmacists, and social workers Work effectively as a member of the interprofessional team Reflect awareness of social determinants of health and their impact on patient care Minimize unnecessary care including tests, procedures, therapies, and ambulatory or hospital encounters	Reflect and learn from critical incidents Understand cost-benefit analysis application to patient care (ie, via principles of screening tests and clinical guidelines) Demonstrate ability to understand and engage in system-level quality improvement in relationship to infectious disease concerns Partner with other health care professionals to identify and propose improvement opportunities within the system in relationship to infectious disease concerns Create and participate in quality improvement initiatives at the institutional or system level

V. Professionalism	
By the end of 3 months, the ID subspecialist ACP will be expected to:	By the end of 6 months, the ID subspecialist ACP will also be expected to:
Demonstrate empathy and compassion toward all patients Maintain appropriate professional relationships with patients, families, and staff Recognize the scope of his/her abilities and ask for supervision and assistance appropriately Treat patients with dignity and respect, regardless of race, culture, gender, ethnicity, age, or socioeconomic status Maintain patient confidentiality	Ensure prompt completion of clinical, administrative, and curricular tasks Recognize disparities in health care among populations and how they may influence patient care

VI. Interpersonal and Communication Skills	
By the end of 6 months, the ID subspecialist ACP will be expected to:	
Provide timely and comprehensive verbal and written communication to patients/advocates after discussion with the ID supervising attending Provide accurate, complete, and timely written communication consistent with medical standards Effectively use an interpreter to engage patients in the clinical setting Demonstrate sensitivity to differences in patients including but not limited to race, culture, gender, sexual orientation, socioeconomic status, literacy, and religious beliefs Deliver appropriate, hypothesis-driven oral presentations Effectively communicate plans of care to all members of the health care team	

Abbreviations : ABGs, arterial blood gases; ACP, advanced care practitioner; CXRs, chest xrays; EKGs, electrocardiograms; ID, infectious diseases; NPV, negative predictive value; PFTs, pulmonary function tests; PPV, positive predictive value.

## INTEGRATION OF MILESTONES AND CORE COMPETENCY ASSESSMENT FOR SUBSPECIALTY ACPS

Milestones are knowledge, skills, attitudes, and other attributes that reflect the development of competence expected for expert consultative practice. The milestones provide a framework for the assessment of the development of the ID subspecialist ACP. The ACP will be rated in each core element of competency [2–4]. These evaluations, based on the suggested time frame outlined in Table 1, are completed by the ID supervising attending physician at the end of the first 3 months, at the end of the first 6 months, and semi-annually thereafter. Bedside rounds are focused on the ACP's ability to develop differential diagnoses and plans of management in conjunction with the care management teams. Direct observation of ACP competencies occurs during these rounds, with feedback encouraged from all health care providers in attendance. In accordance with required ongoing professional practice evaluation (OPPE), quality of patient care is closely monitored by the ID supervising attending, Department of Quality Management, ACP supervisors, and academic department chairs, with timely formative and summative feedback given to the ACP. In a modified 360° review format, peer evaluations are completed by fellow NPs or PAs, nurse managers, residents, and fellows on a biannual basis. Further, the ID ACP has the opportunity to evaluate his/her educational experience including but not limited to the ID supervising attending, other faculty members, residents, fellows, and nurses/other ACPs with whom he/she interacts. The evaluations are confidential, written documents that allow valuable feedback to ensure that the ACP experience is productive. When the curriculum and competency guidelines are first implemented, we suggest that an ID attending physician be designated as the PD with criteria for selection based on ACGME requirements [9, 10] as well as recommendations from the directors of NP and PA training. The program director would oversee the process in conjunction with the ACP and a site-specific coordinator and would ultimately be responsible for signing off on the competency of the ID subspecialty ACP.

## DESCRIPTION OF THE ID SUBSPECIALTY EXPERIENCE

The ID ACP curricular experience will consist of education through direct patient care, didactic sessions, and self-directed learning. Inpatient and/or ambulatory settings will allow the ID ACP subspecialist to refine history and physical examination skills, expand differential diagnostic skills, develop experience in selecting diagnostic tests, and learn to manage a wide variety of infectious diseases. The ID ACP will gain experience working in an interdisciplinary environment. In the inpatient setting, the ID ACP is integrated into the consultation team overseen by a full-time faculty member of the Northwell Division of ID and may include house officers and medical students. ACPs will be assigned patient panels by ID faculty. Members of the ID team examine patients they are following and see new consults requested by any medical service throughout the day. Regular, daily rounds with the ID attending physician will focus on bedside teaching of history and physical examinations. Case presentation skills, analysis of information, and formulation of differential diagnoses and management strategies should reflect “graded responsibility” and expectations. Patient-centered teaching rounds will be supplemented by didactic presentations based on evidence-based practice to ensure up-to-date and comprehensive coverage of a range of infectious diseases. The ACP will communicate with the primary care team or outpatient referring provider all recommendations generated by the consultation. Detailed educational curriculum components, educational objectives, and learning activities associated with educational goals designated by relevant competency are presented in Tables 2 and 3 and are structured around fundamental ACGME IM residency and ID fellowship requirements [9, 10]. These tables outline a blueprint for pathophysiologic and educational competencies as well as the assessment methodologies utilized for the objectives. The ID core lecture series in which the ID “basics,” together with evidence-based review of the literature, are presented will be required of all ACPs either in person or through electronic access. Given the nuances of scheduling, the ACPs will be required to attend all other activities during their shifts. These include the weekly didactic lecture series by local ID faculty focused on additional key topics in ID and weekly ID Grand Rounds, which include practice/guideline updates, case-based presentations with review of the literature, and lectures by invited, nationally prominent individuals with expertise in focused areas of ID.

**Table 2.** Infectious Disease Curriculum Components and Educational Objectives

Conferences and Meetings	
Divisional ID Weekly Conference	Back-to-basics orientation presentations/case-based presentations by members of the ID division, lectures by invited nationally known individuals with expertise in focused areas of ID
Annual Professional Society Meetings	National meetings with ID relevance including but not limited to direct patient care, research, hospital epidemiology, antibiotic stewardship
Site-Specific and Health System Meetings Germane to the Practice of ID	Including but not limited to infection prevention and control, antibiotic stewardship, sepsis task force
GME Events With Relevance to the Practice of ID	Including but not limited to resident report, Medicine Grand Rounds, ACP Grand Rounds

General Principles of Infectious Diseases	
Host Defense Mechanisms	Basic components of the immune system Evaluation of patients with suspected or known immunodeficiency states
Epidemiology and Preventive Medicine	Principles and practices of vaccination in children and adults Role of chemo- or immuno-prophylaxis
Infection Control and Prevention	Basic concepts of infection control, precautions, specific isolation procedures Hospital-associated infections, particularly catheter-related infections, urinary tract infections, wound infections, and pneumonia
Laboratory Testing, Imaging, and General Microbiology	Indications for and interpretation of laboratory tests including PCR, cultures, serology, and other specialized tests Rapid identification of blood culture results and implications for management Interpretation of radiographic studies
Principles of Antimicrobial Therapy	Mechanisms of action, spectrum of activity, pharmacokinetics, and adverse reactions to antibacterial, antiviral, antifungal, and antiparasitic agents Cost of antimicrobial agents Indications for prophylactic antibiotics for selected exposures, conditions, and procedures Antibiotic stewardship and de-escalation of therapy Outpatient intravenous antibiotic therapy

Medical Knowledge of Major Clinical Syndromes: Epidemiology, Diagnosis, Clinical Course, Treatment, and Prevention	
Fever	Etiologies of infectious and noninfectious causes of fever
Bloodstream Infections	Etiologies including gram-positive and gram-negative infections with special consideration of *S. aureus,* fungi, and multidrug-resistant organisms
Central Nervous System Infections	Meningitis, encephalitis, and related infections Etiologies including bacterial/borrelial, viral, mycobacterial, fungal, or parasitic Indications for lumbar puncture and interpretation of CSF findings
Respiratory Infections	Upper respiratory tract infections, including sinusitis, otitis, epiglottitis, odontogenic infections, viral syndromes Bronchial and pleuropulmonary infections including community-acquired, aspiration- and hospital/ventilator-associated pneumonia; lung abscess, empyema, atypical mycobacteria, and TB; SARS, influenza, other newer entities
Cardiovascular and Endovascular Infections	Native and prosthetic valve endocarditis, myocarditis, pericarditis, thrombophlebitis Infections of implantable devices, endovascular grafts, and other intravascular devices
Skin and Soft Tissue Infections and Related Conditions	Cellulitis, erysipelas, impetigo, dermatophytosis, and necrotizing infections Adverse drug reactions Diabetic foot infections including medical/surgical treatment and prevention strategies
Bone and Joint Infections	Bacterial, viral arthritis Lyme disease Rheumatic fever Acute and chronic osteomyelitis Interpretation of imaging Indications for biopsy, aspiration, or surgery
Gastrointestinal Tract Infections	Peritonitis, appendicitis, cholecystitis, pancreatitis, enteritis, intra-abdominal abscess, food poisoning
Genitourinary Tract Infections	Cystitis, complicated UTI, and pyelonephritis Asymptomatic bacteriuria Pelvic inflammatory disease, pelvic abscess, endometritis Epididymitis/orchitis/prostatitis
Infections of Prosthetic Devices	Prosthetic joint or other hardware infection Ventricular shunt infections Mesh infections Implant infections of other sites
Infections Related to Trauma	Human and animal bites Rabies postexposure prophylaxis
Infections of the Eye	Bacterial and viral conjunctivitis/keratitis Endophthalmitis
Sepsis Syndromes	Current understanding of the immunologic cascade, proinflammatory cytokines, and the role of endotoxin Modalities of care including selection of antibiotics, supportive measures, and adjunctive/immunomodulatory therapies
Hospital-Associated Infections	Infections associated with intravascular access devices, foreign bodies, and stents Respiratory infections associated with mechanical ventilation Antibiotic-associated colitis Catheter-related urinary tract infections Surgical wound infections
Sexually Transmitted Infections	Gonorrheal infections, nongonococcal urethritis, and mucopurulent cervicitis Genital ulcer disease including herpes, syphilis, and chancroid Pelvic inflammatory disease and tubo-ovarian abscess Vaginitis STI-related prostatitis, epididymitis, orchitis
Infections in Travelers	Pretravel counseling, vaccination, and prophylaxis Malaria Dengue Other common and/or important syndromes and pathogens
Granulocytopenic Patients	Empiric antibiotic use in febrile neutropenia Assessing risk of bacterial, viral, and fungal infections Role of prophylactic antimicrobials and colony-stimulating factors Principles of precautions
Impact of Immunosuppressive Medications	Adverse effects, medication interactions, and potential complications of commonly used immunosuppressive or immunomodulatory agents
Infections in Transplant Patients	Post-transplant infectious complications Graft-vs-host disease and other complications Prophylactic antimicrobial strategies
HIV/AIDS	Evaluation and risk factors Opportunistic infections and prophylaxis Antiretroviral treatment and monitoring Pre- and postexposure prophylaxis
Infections in Pregnancy	Infectious diseases with specific issues related to pregnancy such as CMV, toxoplasmosis, or erythrovirus/parvovirus Prevention of group B *Streptococcus* infection Evaluation of peripartum fever including chorioamnionitis, endometritis, pelvic thrombophlebitis, mastitis Safety of antibiotics in pregnancy

Abbreviations: ACP, advanced care practitioner; CMV, cytomegalovirus; CSF, cerebrospinal fluid; ID, infectious diseases; PCR, polymerase chain reaction; SARS, severe acute respiratory syndrome; STI, sexually transmitted infection; TB, tuberculosis; UTI, urinary tract infection.

**Table 3.** Principal Educational Goals by Relevant Competency

I. Patient Care	
Principal Educational Goals	Learning Activities
Enhance skills in obtaining patient history, encompassing all organ systems relevant to the infection being evaluated	AR, DPC, SS
Perform a thorough physical examination	AR, DPC, SS
Generate appropriate differential diagnoses	AR, DPC, IDGR, SS
Write notes appropriate to the ID consultant that communicate clearly and effectively diagnostic possibilities/suggestions	AR, DPC
Develop evidence-based management strategies as an ID subspecialist	AR, DPC, IDGR, SS

II. Medical Knowledge (See Above “Medical Knowledge Regarding Major Clinical Syndromes”)	
Principal Educational Goals	Learning Activities
Develop a sound knowledge base in the disease-oriented topics described in the curriculum	AR, DPC, IDGR, SS
Integrate clinical knowledge with a sound understanding of disease pathophysiology	AR, DPC, IDGR, SS

III. Practice-Based Learning and Improvement	
Principal Educational Goals	Learning Activities
Analyze performance during the rotation and identify areas of improvement	AR, DPC, IDGR, SS
Be receptive to constructive feedback, develop strategies for improvement, and assess performance outcomes	AR, DPC, SS
Use information technology to retrieve applicable data, analyze the information, and apply it to patient care	AR, DPC, IDGR, SS

IV. Interpersonal and Communication Skills	
Principal Educational Goals	Learning Activities
Develop an appreciation for and skill in conveying complex information regarding infectious processes	AR, DPC, SS
Develop an appreciation for and skill in discussing with empathy and sensitivity the diagnosis of an infectious process with patients	AR, DPC, SS
Communicate effectively with family members and work closely with health care proxies when indicated	AR, DPC, SS

V. Professionalism	
Principal Educational Goals	Learning Activities
Treat patients with respect, compassion, dignity, and integrity	AR, DPC, SS
Demonstrate sensitivity and responsiveness to patients’ gender, age, culture, religion, sexual preference, socioeconomic status, beliefs, behaviors, and disabilities	AR, DPC, SS
Strive to have your conduct serve as a role model for students and junior providers	AR, DPC, SS

VI. Systems-Based Practice	
Principal Educational Goals	Learning Activities
Learn how to serve in the role of consultant and work effectively with the primary care physician and other providers	AR, DPC, IDGR, SS
Effectively incorporate consultants into patient care	AR, DPC, IDGR, SS
Understand the importance of all staff involved in patient care	AR, DPC, IDGR, SS
Understand the impact of the types of health insurance on home services, particularly home antibiotic therapy	AR, DPC, IDGR, SS

Abbreviations: AR, attending rounds; DPC, direct patient care; ID, infectious diseases; IDGR, ID Grand Rounds; SS, self study.

## DISCUSSION

The ACGME common program requirements describe a basic set of standards that must be met in order to graduate a resident or fellow from a training program in the United States [9, 10]. These requirements “set the context within clinical learning environments for development of the skills, knowledge, and attitudes necessary to take personal responsibility for the individual care of patients. In addition, they facilitate an environment where residents and fellows can interact with patients under the guidance and supervision of qualified faculty members who give value, context, and meaning to those interactions” [9]. The ACGME requirements provide the basis for the design of our proposed ACP curriculum and competency guidelines.

In response to the Institute of Medicine recommendation in 2011 that nursing education move to a CBE framework [27], NP core competencies were developed to ensure that NPs graduate with the knowledge, skills, and abilities that are essential to competent clinical practice. Chan et al. refined the core competencies initially put forth by the NONPF and AACN for trainees in BSN-DNP programs using a CBE platform [13]. Wu et al. developed and validated a scale to measure core competency achievement specifically in reference to an ID nurse specialist [14].

The NCCPA coordinated an effort along with 3 other national organizations to define PA competencies [15]. Postgraduate programs for Pas, which began in 1973, expanded to about 72 programs by 2020, encompassing a wide range of medical and surgical disciplines. Interestingly, for many reasons, not all these programs are ARC-PA accredited. Twelve months is the average program length, but <1% of PA graduates attend a postgraduate program [28]. Although the NCCPA confers various discipline-specific certificates of added qualification, none exists for ID [29]. In addition, the website of the Association of Postgraduate Physician Assistant Programs does not list any PA or joint PA/NP postgraduate programs specific to ID [25]. To our knowledge, there are also no ID fellowship programs for NPs alone in the United States.

At the MD Anderson Cancer Center, inpatient and outpatient responsibilities were put into place based on recommendations from the American Society of Clinical Oncology to expand the workforce with midlevel providers. Part of this initiative included participation in a multidisciplinary antimicrobial stewardship program in the intensive care unit. The consensus was that most of the PAs in ID had received “an abbreviated level of ID education during their formal training” and that “most of their clinical knowledge is acquired during on-the-job training,” with no formal curriculum or competency requirements available at that time [30]. Despite the lack of a formal curriculum, use of ACPs in ID has resulted in improvements in health care efficiency. Specifically, time to consultation and length of stay both decreased after Pas were integrated into the ID consult service [31].

Gail et al. (in 2004) broadly outlined a curriculum at the Master's level for clinical nurse specialists and NPs entering the field of ID. The curriculum was divided into a core curriculum including courses in epidemiology, microbiology, immune response, ID nursing, pharmacology, culturally competent care, and nursing research methods. This was followed by further course work depending on the practitioner's anticipated role. For adult ID NPs, these courses included clinical assessment and management, diagnostic testing, decision-making, health promotion/disease prevention, and ID medicine [32].

Training programs for ACPs within a focused area in ID have been described. Most of these programs relate to HIV care either as a stand-alone effort or integrated into a general curriculum [33–36]. Hayes et al. reviewed the impact of integrating coursework on HIV care into NP training programs. Their curriculum was integrated into an NP primary care curriculum [33]. McGee et al. at Duke implemented a training program for NPs providing primary care to persons with HIV. This included extensive clinical supervision as well as didactic work [34]. Farley's group at Johns Hopkins developed a curriculum that integrated HIV prevention, treatment, and care in the adult/geriatric NP program. NPs were assigned to spend 50% of their time in primary care and 50% of their time in HIV-focused care [36]. All of these training programs, however, have not been targeted to train ACPs to provide practice over the full spectrum of ID.

There remains a dearth of literature addressing formal curriculum and competency for ACPs who are in a subspecialty IM practice. Integration of well-trained ACPs with residents and fellows promotes collaboration and strengthening of the academic practice partnership [6–10]. Our document provides the foundation for the scope of practice with an emphasis on clinical experience as the principal means of achieving competence while continuing to be guided and supervised in the process. To our knowledge, there are no curriculum or competency guidelines for ACPs to provide the full spectrum of ID practice. The proposed curriculum and competency guidelines utilize an interprofessional approach to include the proposed elements required for achievement of competence. In addition, our proposal greatly adds to the available literature for ACPs who have finished training and are now pursuing a career as an ID subspecialist and, with modification, may be applicable to other IM subspecialties.
